# Smoking and attention in schizophrenia spectrum disorders: What are we neglecting?

**DOI:** 10.3389/fpsyg.2023.1114473

**Published:** 2023-03-30

**Authors:** Barbora Keřková, Karolína Knížková, Aneta Siroňová, Aleš Hrubý, Monika Večeřová, Petra Šustová, Juraj Jonáš, Mabel Rodriguez

**Affiliations:** ^1^National Institute of Mental Health, Klecany, Czechia; ^2^Department of Psychiatry, First Faculty of Medicine, Charles University and General University Hospital, Prague, Czechia; ^3^Department of Psychology, Faculty of Social Studies, Masaryk University, Brno, Czechia; ^4^Department of Psychology, Faculty of Arts, Charles University, Prague, Czechia

**Keywords:** smoking, nicotine, schizophrenia, psychotic disorder, first-episode, self-medication, attention, CPT

## Abstract

**Introduction:**

Individuals with schizophrenia spectrum disorders (SSDs) record elevated rates of smoking, which is often attributed to their effort to self-medicate cognitive and attentional symptoms of their illness. Empirical evidence for this hypothesis is conflicting, however. In this study, we aimed to test predictions derived from the cognitive self-medication hypothesis. We predicted that cigarette smoking status and extent would predict the attentional performance of participants with SSDs. Simultaneously, we wished to address methodological gaps in previous research. We measured distinct attentional components and made adjustments for the effects of other, attention-modulation variables.

**Methods:**

Sixty-one smokers (82.0% males, 26.73 ± 6.05 years) and 61 non-smokers (50.8% males, 27.10 ± 7.90 years) with recent-onset SSDs completed an X-type Continuous Performance Test, which was used to derive impulsivity and inattention component scores. Relationships between the two component scores and cigarette smoking status and extent were assessed using hierarchical regression. Effects of estimated premorbid intellectual functioning and antipsychotic medication dosage were held constant.

**Results:**

Smokers had significantly higher inattention component scores than non-smokers when covariates were controlled (*p* = 0.026). Impulsivity remained unaffected by smoking status (*p* = 0.971). Cigarette smoking extent, i.e., the number of cigarettes smoked per day, was not associated with either inattention (*p* = 0.414) or impulsivity (*p* = 0.079).

**Conclusion:**

Models of smoking-related attentional changes can benefit from the inclusion of sample-specific component scores and attention-modulating covariates. Under these conditions, smokers with SSDs can show a partial attentional benefit. However, the limited scope of this benefit suggests that the cognitive self-medication hypothesis requires further testing or reconsidering.

## Introduction

The prevalence of smoking among people with schizophrenia spectrum disorders (SSDs) is generally estimated to be between 60 and 70%, which is significantly higher than the rates seen in other psychiatric conditions or the general population (Ziedonis et al., 2008; Chapman et al., 2009; Zeng et al., 2020). This increased prevalence is often explained in terms of the cognitive self-medication hypothesis, which posits that individuals with schizophrenia start and maintain tobacco use for its ameliorating effects on some of their cognitive deficits, particularly attentional deficits (Ziedonis et al., 2008; Winterer, 2010; D’Souza and Markou, 2012; Manzella et al., 2015). While the hypothesis has attained widespread use in research and clinical practice, the evidence for it is lacking (Winterer, 2010; Manzella et al., 2015).

One of the primary active compounds in tobacco smoke is nicotine. As a person smokes, nicotine is absorbed into the blood stream and quickly enters the brain. There, nicotine binds to nicotinic cholinergic receptors, leading to increased activity in the prefrontal cortex, thalamus, and visual system, and the release of several neurotransmitters, including dopamine (Benowitz, 2009). Nicotine may also counteract the effects of some forms of antipsychotic treatment by partially restoring the medication-induced blockage of dopamine receptors (Winterer, 2010). Correspondingly, smokers with schizophrenia can show reduced plasma levels of antipsychotic medications (Winterer, 2010). These observations have previously given rise to one of several self-medication hypotheses that have circulated schizophrenia research, namely that patients with SSDs gravitate toward tobacco use to diminish the side effects of their medication (Winterer, 2010; Manzella et al., 2015).

However, the antipsychotic version of the self-medication hypothesis had limited success, as subsequent research could not substantiate it. For example, pertinent side effects such as extrapyramidal symptoms were found to occur in as many smokers as non-smokers with schizophrenia (Manzella et al., 2015). Moreover, a high prevalence of smoking was documented in first-episode and prodromal patients, suggesting that it precedes the disorder (Vaňurová, 2000; Riala et al., 2005). Ultimately, the hypothesis that individuals with SSDs smoke to relieve medication side effects was considered insufficient to explain the elevated rate of smoking in the population.

The cognitive self-medication hypothesis also arose from initially promising findings. For example, early neurophysiological research suggested that nicotine normalized deficits in sensory processing, including auditory sensory gating (Adler et al., 1993) or prepulse-inhibition (Kumari et al., 2001). Furthermore, evidence from animal models and human imaging studies indicated that smoking could improve attention and vigilance, possibly by promoting increased activation in prefrontal areas (Winterer, 2010). While the resulting cognitive self-medication hypothesis has become a widely cited reason for the increased prevalence of smoking in SSDs, it has also not gone uncontested. In their review, Manzella et al. (2015) sought evidence for several predictions derived from it, but with limited success. While the authors suggested that the neural basis for the hypothesis was substantial, there were plentiful inconsistencies “to give us pause in continuing to accept the hypothesis without question” (Manzella et al., 2015: 43). Another review by Winterer (2010: 116) concluded that smoking in schizophrenia was “to some considerable extent” explained by self-medication of clinical symptoms. However, the review also highlighted some of the shortcomings of the hypothesis. In particular, direct behavioral evidence for it was sparse or conflicting. Moreover, the cognitive benefits of smoking and nicotine in schizophrenia seemed limited to a few neuropsychological tests.

The Continuous Performance Test (CPT) is one of the most studied neuropsychological tests in relation to smoking in SSDs (see, e.g., Wing et al., 2011; Hahn et al., 2012; Roth et al., 2013). During this test, participants must quickly respond to certain types of stimuli, while withholding their response to all the others. Several different types of the test have been used in the present context. In the X-type CPT (Conners, 2000; Conners, 2014), participants respond to all the stimuli other than the infrequently presented letter ‘X’. In the IP-type CPT (Cornblatt et al., 1988), participants withhold their response to all the stimuli other than two identical stimuli presented in sequence. Other variants include the CPT-O (in which participants respond to the letter ‘O’) or the CPT-OX (in which they respond to an ‘O’ only if preceded by an ‘X’).

The CPT produces upward of 12 scores, which contribute to four attentional domains: inattention, impulsivity, sustained attention, and vigilance. The former two domains have been the focus of studies on attention in smokers with SSDs, with the most frequently analyzed scores including hit reaction times (RTs), errors of omission, and errors of commission. Hit RTs measure the speed of accurate responses. Omissions arise when no response is given for targets, and commissions arise when a response is given for non-targets. Other measures include overall accuracy, detectability (which measures the difference between target and non-target distributions), perseverations (trials with RTs that are too short (< 100 ms) to relate to the stimulus), and variability of standard error (which measures RT consistency throughout the test’s duration).

At least three studies documented improved CPT performance among smokers with SSDs. Wing et al. (2011) reported a significant association between schizophrenia patients’ smoking history and CPT-IP accuracy, commission rate, and variability, while hit RTs did not appear to be affected. In another study, heavy smokers with first-episode psychosis recorded marginally faster RTs on CPT-O and CPT-OX but comparable commission rates on both (Zabala et al., 2009). A significantly reduced rate of errors of omission on the CPT-OX was also observed, which was later confirmed in a follow-up to this study (Segarra et al., 2011).

Other studies found no difference between smokers and non-smokers with SSDs or even impaired performance in the former group. In one study, smokers and non-smokers with schizophrenia recorded comparable CPT-IP accuracy scores, hit RTs, and detectability scores (Hahn et al., 2012). A large-scale study corroborated that accuracy was unaffected by smoking in first-episode psychosis (Hickling et al., 2018). A recent study also found no significant differences in CPT-IP performance between smokers and non-smokers with chronic schizophrenia (Wang et al., 2023). Finally, Roth et al. (2013) documented worse CPT-IP and CPT-X performance in smokers with SSDs. Substantial between-group differences were detected in CPT-IP, where all detectability, accuracy, hit RT SD, and omission rates showed a deficit. On the CPT-X, commission rates were elevated in smokers, while all other scores were comparable between the two groups.

The aforementioned studies indicate conflicting evidence in terms of attentional benefits of *ad-libitum* smoking in SSDs. This inconsistency is indicative of shortcomings, which could involve methodological issues or the cognitive self-medication hypothesis itself. For example, the most frequently analyzed CPT scores pertain to inattention and impulsivity. Yet, only a few studies utilize the CPT-X, which is considered to be better suited for the assessment of these domains (Conners, 2000; Riccio et al., 2002; Roth et al., 2013). As Hahn et al. (2012) noted, studies tend to forego a comprehensive CPT analysis in favor of a more targeted analysis of a few test scores, which could skew the results and their subsequent interpretation. The CPT produces too many scores to allow for a meaningful analysis of each, and neither can it yields a grand average. This suggests that the use of data-derived factor or component scores should be the preferred means of analysis (Egeland and Kovalik-Gran, 2010a). Extraneous variables also require closer consideration. For example, the type and dosage of antipsychotic medication can be related to patient performance in several domains of cognition, including visual attention (Hori et al., 2006; MacKenzie et al., 2018; Fernandes et al., 2019). Therefore, it may be suitable to adjust for medication use in statistical models of smoking and attention in SSDs. Premorbid intellectual functioning should also be more carefully considered. Premorbid IQ and educational attainment tend to be lower among smokers in both general and psychiatric populations (Huisman et al., 2005; Ziedonis et al., 2008; Wing et al., 2011; Hidese et al., 2019). Estimates of lifetime intellectual, social, or occupational functioning, which can sometimes be grouped under the concept of the cognitive reserve, are also positively related to cognitive performance in individuals with SSDs (Herrero et al., 2020; Rodriguez et al., 2022).

Given the outlined inconsistencies in previous findings, it may also be possible that the cognitive self-medication hypothesis is inaccurate or incomplete. This being the case could have important clinical implications as the hypothesis may dissuade clinicians from promoting abstinence in their patients (Ziedonis et al., 2008). Yet, smoking clearly affects physical health, contributing to a range of pathologies that reduce life quality and increase mortality (Hennekens, 2007; Benowitz, 2009; Kelly et al., 2011). Individuals with SSDs appear to be particularly prone to smoking-related cardiovascular disease, which accounts for 75% of all deaths in this population and contributes to a significantly reduced life expectancy (Hennekens, 2007; Kelly et al., 2011).

The present study aimed to test a key prediction that can be derived from the cognitive self-medication hypothesis, i.e., that patients with SSDs who smoke would show better cognitive performance than those who do not (Manzella et al., 2015). In particular, we hypothesized a positive relationship between *ad-libitum* smoking and CPT performance (H1), and between cigarette smoking extent and CPT performance (H2). Simultaneously, we wished to account for several of the methodological gaps identified above. To this end, we opted for an X-type CPT and analyzed the component scores derived from it. We also considered the contribution of two extraneous variables, i.e., antipsychotic medication dosage and estimated intellectual premorbid functioning. Additionally, we only selected participants with recent-onset SSDs (i.e., their first diagnosis of schizophrenia or acute and transient psychotic disorder) for this study. This reduced the likelihood that the variations observed between smokers and non-smokers with the disease were confounded by other, chronicity-related factors, such as prolonged institutionalization, depression, or social isolation. Finally, to aid interpretation and provide additional context, we explored relationships between smoking and the clinical characteristics of the sample, including symptom severity and general functioning.

## Materials and methods

Data for this study were extracted from the “Early Schizophrenia Outcome” (ESO) project conducted at the National Institute of Mental Health in Klecany, Czech Republic (NIMH-CZ). The project involves the creation of a multimodal, nationwide database, which collects data on individuals following their first SSD episode. This study analyzed data from the clinical and neuropsychological assessments conducted within the project. Additional neuropsychological results from the ESO project were described in, e.g., Rodriguez et al. (2019), Hájková et al. (2021), and Rodriguez et al. (2022). The ESO study conformed to the ethical standards described in the latest version of the Declaration of Helsinki, and was approved by the NIMH-CZ Research Ethics Board (protocol number: 127/17). Written informed consent was provided by each participant or their legal guardian.

### Participants

A total of 122 patients with an ICD-10 diagnosis of schizophrenia (F20.x) (*N* = 65, 53.3%) or acute and transient psychotic disorder (F23.x) (*N* = 57, 46.7%) were included in this study. The participants were mostly male (*N* = 81, 66.4%), aged 16–45 (*M* = 26.91, *SD* = 7.01), with 10–23 years of education (*M* = 14.88, *SD* = 3.24).

Participants were excluded if they reported a history of brain trauma or a pre-existing diagnosis of a disorder of childhood development or learning (e.g., ADHD, dyslexia). Additional grounds for exclusion included: meeting the criteria for affective psychosis (e.g., schizoaffective disorder or bipolar disorder), recent (< 12 months) neuropsychological assessment, missing data on smoking or CPT performance, and signs of invalid CPT administration (i.e., T-scores >100 on the CPT Omissions scale, see, e.g., Erdodi et al., 2018). Participants were invited into the study after their first psychotic episode and once they were symptomatically stabilized according to their supervising clinician. Two participants were not receiving any medication at the time of their participation. The remaining 120 were all using atypical antipsychotics in the form of monotherapy (*N* = 82, 68.3%) or antipsychotic polytherapy (*N* = 38, 31.7%). Table 1 describes additional clinical characteristics of the sample.

**Table 1:** Clinical characteristics in smokers (*N* = 61) and non-smokers (*N* = 61) with recent-onset SSDs, and their relationship to cigarette smoking status and extent.

	Smokers	Non-smokers	Between-group	Within-smokers
Variable	*M ± SD* or *N* (%)		*p*	
CPZ EKVI (mg)	414.85 ± 198.26	348.28 ± 153.05	0.041	0.246
DUP (months)	3.54 ± 6.15	2.41 ± 4.46	0.549	0.915
PANSS-positive	10.87 ± 3.57	10.25 ± 3.03	0.301	0.928
PANSS-negative	15.74 ± 5.64	16.02 ± 5.31	0.779	0.142
PANSS-general	27.79 ± 7.16	28.69 ± 6.77	0.476	0.628
CGI-S	3.30 ± 1.28	3.36 ± 1.10	0.762	0.387
GAF	65.60 ± 14.83	66.21 ± 15.43	0.821	0.449
Diagnosis			0.856	0.524
F20.x	33 (52.5%)	32 (54.1%)		
F23.x	28 (47.5%)	29 (45.9%)		

SSDs, schizophrenia spectrum disorders, CPZ EKVI, antipsychotic medication dosage in chlorpromazine equivalents; DUP, duration of untreated psychosis; PANSS, the Positive and Negative Syndrome Scale, CGI-S, the Clinical Global Impression Severity Scale; GAF, the Global Assessment of Functioning Scale.

### Measures and procedure

#### Smoking history

Participants categorized themselves as smokers or non-smokers with respect to the last 12 months. Smokers were also asked to state the number of cigarettes they smoked per day (termed “cigarette smoking extent” from here onward). Note that neither the extent nor the total (lifetime) duration of smoking were considered as additional criteria for inclusion in the smoker group. Participants were not nicotine-deprived during the duration of the study, and they were allowed to smoke *ad-libitum*.

#### Attentional performance

Attentional performance was measured using an X-type CPT: Conners’ Continuous Performance Test II (Conners, 2000). The test was administered as part of a wider neuropsychological assessment, which took 150 min to complete in total. CPT administration took place toward the end of this assessment and after a short break. Participants were seated in front of a computer and instructed to press the left mouse key or space bar as quickly as possible after the presentation of any letter other than ‘X’. The standard paradigm consists of 18 blocks of 20 trials each, i.e., 360 trials in total. Within each trial, a letter was presented for 250 ms and was followed by a variable 1–4 s inter-stimulus interval. The test took 14 min to complete, excluding the practice trials. The CPT yields several reverse-scored T-scores. Six were considered for analysis: hit RT, errors of omission, errors of commission, detectability, perseverations, and variability. The scores were z-transformed and (re-)reversed-scored, so that higher scores corresponded to better functioning in the given area.

#### Other measures

Chlorpromazine equivalents of antipsychotic medication dosage were obtained following standard guidelines (Gardner et al., 2010). Premorbid intellectual functioning was estimated from the participants’ years of education and their raw scores on the Information subtest from the Wechsler Adult Intelligence Scale, Third Revision (WAIS-III). The two variables were *z*-transformed and averaged. Years of education are typically correlated with IQ and can be used to roughly estimate its premorbid levels (Matarazzo and Herman, 1984). The WAIS-III Information subtest is thought to belong to the category of “hold” subtests (Wechsler, 1997; Lezak et al., 2012), which reflect premorbid abilities and crystallized intelligence (Ryan et al., 2000). The rationale is that cognitive skills related to semantic knowledge tend to be less affected by pathology and tend to remain preserved even after the onset of a disorder.

Several clinical characteristics were used to describe the sample and to explore their potential relationship with the sample’s smoking history. These clinical characteristics included the duration of untreated psychosis (DUP), the Positive and Negative Syndrome Scale (PANSS) (Kay et al., 1987), the Clinical Global Impression Severity Scale (CGI-S) (Guy, 1976), and the Global Assessment of Functioning (GAF) (American Psychiatric Association, 1994). DUP is the difference between the time when first psychotic symptoms appeared and the time when antipsychotic treatment was initiated. This measure was log-transformed prior to analysis to account for its skewness. PANSS assesses the severity of positive (7 items), negative (7 items), and general psychopathology symptoms (16 items). Each item is assessed on a 7-point Likert scale, with higher scores corresponding to higher symptom severity. The CGI-S measures illness severity on a 7-point scale ranging from 1 (normal) to 7 (among the most severely ill patients). GAF is used to subjectively rate social, occupational, and psychological functioning. It is a numeric scale, ranging from 100 (extremely high functioning) to 1 (severely impaired).

### Statistical analysis

Statistical analysis was conducted in SPSS v. 28.0. The significance level was set at *p* < 0.05. Between-group differences in demographics, clinical characteristics, and estimated premorbid intellectual functioning were probed by means of a two-tailed, independent-samples *t*-test (Student’s or Welch’s, as relevant) or *χ*^2^ test. Within the smoker group, associations between cigarette smoking extent and clinical characteristics were explored *via* Spearman’s correlation.

Since the CPT’s internal structure is equivocal and possibly dependent on a test-taker’s psychiatric status (Conners, 2000; Egeland and Kovalik-Gran, 2010a; Egeland and Kovalik-Gran, 2010b), we performed principal components analysis of the six CPT scores. Components were extracted based on scree plot and eigenvalue criteria. Since the components that were initially extracted were not correlated, an orthogonal rotation using the varimax method was applied. Two component scores were derived using the regression method. They were uncorrelated.

Relationships between cigarette smoking status and the component scores (H1) were assessed in hierarchical regression analysis. Assumptions for the two tests were checked using the variance inflation factor, the Durbin-Watson test, and by visual inspection. No problems were noted. Smoking status was entered in step 1, with smokers coded as 1, and non-smokers as 0. Covariates were estimated premorbid intellectual functioning and antipsychotic medication dosage in chlorpromazine equivalents. Both were added in step 2 as z-scores (forced entry method), meaning that the resulting regression coefficients can be considered partially standardized. Significant effects were probed further by adding relevant product terms in step 3.

A similar procedure was applied to assess the relationships between cigarette smoking extent (z-transformed) and CPT component scores (H2). These two regression models were only constructed for the smoker group. Assumptions for the analysis were met. Cigarette smoking extent was entered in step 1 and covariates in step 2.

## Results

### Smoking and sample characteristics

Sixty-one (50.0%) of the participants were smokers. Their cigarette smoking extent was varied: they smoked 11.31 ± 6.82 cigarettes per day, on average.

There were 50 (82.0%) males in the smoker group, compared to 31 (50.8%) among non-smokers. This difference was statistically significant, *χ*^2^ (1, *N* = 122) = 13.262, *p* < 0.001. Smokers (*M* = 26.73, *SD* = 6.05) and non-smokers (*M* = 27.10, *SD* = 7.90) did not differ in age (*p* = 0.773, *d* = 0.052).

Medication dosage was higher among smokers, *t*(121.20) = −2.062, *p* = 0.042, *d* = −0.373. Other clinical characteristics did not differ between smokers and non-smokers (Table 1: Between-groups). None were associated with the extent of cigarette smoking within the smoker group (Table 1: Within-smokers).

In smokers, estimated premorbid intellectual functioning was slightly below the sample’s mean (*M* = −0.20, *SD* = 0.83), while non-smokers scored higher (*M* = 0.20, *SD* = 0.83). This difference was statistically significant, *t*(129) = 2.619, *p* = 0.010, *d* = 0.474. Within the smoker group, cigarette smoking extent and estimated premorbid intellectual functioning were not significantly related (*p* = 0.704).

### Principal components analysis

Sampling was acceptable (KMO = 0.603) and inter-item correlations were sufficiently large, *χ^2^* (15) = 338.633, *p* < 0.001. Communalities were the lowest for perseverations at 0.484 and otherwise ranged between 0.628 and 0.883. Following the inflections observed in the scree plot as well as Kaiser’s eigenvalue criteria, two components were retained, which together explained a total of 72.01% of the variance observed. Table 2 shows the loadings after rotation. Component 1 was labeled as Impulsivity, being predominantly composed of errors of commissions, detectability, and *fast* RTs. Component 2 received loadings from errors of omissions, variability, and perseverations, and was therefore labeled Inattention. Of note, *slow* RTs contributed to Inattention in the initial solution with a loading of 0.532, but this subsided substantially after rotation (Table 2).

**Table 2:** The orthogonally rotated solution to the principal components analysis of CPT scores.

	Loadings	
Score	Component 1: Impulsivity	Component 2: Inattention
Commissions	**0.901**	0.267
Hit RT	**−0.863**	0.177
Detectability	**0.846**	0.135
Variability	−0.141	**0.895**
Omissions	0.144	**0.779**
Perseverations	**0.440**	**0.539**
Eigenvalue	2.709	1.616
% of variance	45.16	26.94

Component loadings >0.400 appear in bold.

### Cigarette smoking status and CPT component scores

#### Inattention

**Figure 1:**
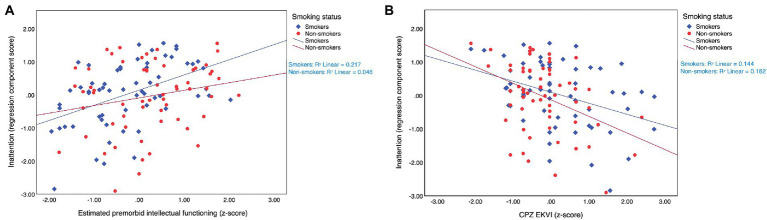
Estimated premorbid intellectual functioning **(A)** and antipsychotic medication dosage **(B)** affected inattention component scores approximately equally in smokers (*N* = 61) and non-smokers (*N* = 61) with SSDs. SSDs, schizophrenia spectrum disorders; CPZ EKVI, antipsychotic medication dosage in chlorpromazine equivalents.

Table 3 describes the two models that were constructed for the inattention component score. In model 1, smoking status was not a significant predictor of inattention, although the component scores obtained by smokers (*M* = 0.038, *SD* = 0.963) tended to be higher than those of non-smokers (*M* = −0.038, *SD* = 1.043). This initial between-group difference of 0.076 increased to 0.372 in model 2, in which covariate effects were held constant. In this model, smoking status predicted inattention component scores with statistical significance. As a whole, the model explained 25.5% of the variance observed in inattention. The partial effects of the covariates were relatively substantial, such that higher estimated premorbid intellectual functioning predicted higher inattention scores, while higher antipsychotic medication dosage related to worse performance. Interaction effects of group and estimated premorbid intellectual functioning (*B* = 0.136, 95% CIs: −0.189-0.460, *t* = 0.827, *p* = 0.410) and group and antipsychotic medication dosage (*B* = 0.249, 95% CIs: −0.084-0.582, *t* = 1.480, *p* = 0.141) were non-significant. This suggested that the covariates contributed to inattention approximately equally in both groups, as can also be seen from the simple slopes in Figure 1.

**Table 3:** Smoking status was a significant predictor of inattention when the effects of covariates were held constant, but impulsivity remained unaffected.

	Inattention^a^			Impulsivity^b^		
Model and predictor	*B* [95% CI]	*t*	*p*	*B* [95% CI]	*t*	*p*
**Model 1**						
Smoking status	0.076 [−0.292–0.217]	0.416	0.678	−0.087 [−0.447–0.272]	−0.481	0.632
**Model 2**						
Smoking status	0.372 [0.026–0.044]	2.249	0.026	0.007 [−0.367–0.381]	0.036	0.971
EPIF	0.335 [0.173–0.497]	4.103	<0.001	0.133 [−0.052–0.318]	1.425	0.157
CPZ EKVI	−0.382 [−0.543–-0.222]	−4.730	<0.001	−0.088 [−0.271–0.095]	−0.953	0.342

For smoking status, smokers were coded as 1, non-smokers as 0. EPIF, estimated premorbid intellectual functioning; CPZ EKVI, antipsychotic medication dosage in chlorpromazine equivalents. Both variables were entered into the model as z-scores. ^a^Model 1: *F*(1, 120) = 0.173, *p* = 0.678, *R*^2^ = 0.001; Model 2: *F*(3, 118) = 13.472, *p* < 0.001, *R*^2^ = 0.255; Δ*R*^2^ = 0.254, *p* = 0.001. ^b^Model 1: *F*(1, 120) = 0.231, *p* = 0.632, *R*^2^ = 0.002; Model 2: *F*(3, 118) = 1.080, *p* = 0.360, *R*^2^ = 0.027; Δ*R*^2^ = 0.025, *p* = 0.226.

#### Impulsivity

With regard to impulsivity, smokers obtained component scores that were just below the sample’s mean (*M* = −0.044, *SD* = 0.838), while non-smokers tended to perform better (*M* = 0.044, *SD* = 1.145). Nonetheless, both models that were constructed for the impulsivity component score returned non-significant findings, meaning that neither smoking status, nor the covariates affected it (Table 3).

### Cigarette smoking extent and CPT component scores

#### Inattention

**Figure 2:**
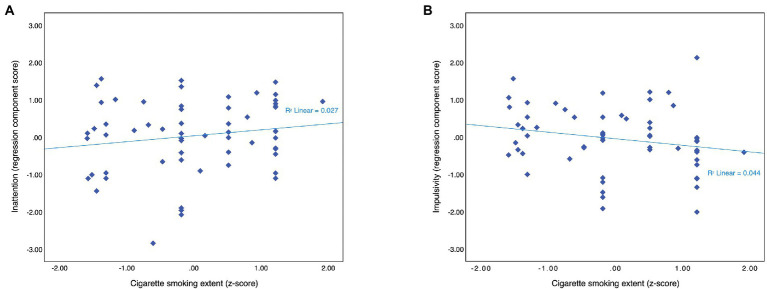
Among smokers (*N* = 61), cigarette smoking extent did not affect either inattention component scores **(A)** or impulsivity component scores **(B)**.

There was a non-significant, positive relationship between cigarette smoking extent and the inattention component score (Figure 2A). Entering estimated premorbid intellectual functioning and antipsychotic medication dosage into the model led to a significant model fit which explained a total of 32.9% of the variance observed, but the effect of the cigarette smoking extent remained non-significant (Table 4).

**Table 4:** Among smokers with SSDs (*N* = 61), cigarette smoking extent and CPT component scores were not significantly related.

	Inattentiveness^a^			Impulsivity^b^		
Model and predictor	*B* [95% CI]	*t*	*p*	*B* [95% CI]	*t*	*p*
**Model 1**						
Smoking extent	0.159 [−0.088 to 0.407]	1.289	0.202	−0.176 [−0.390 to 0.037]	−1.652	0.104
**Model 2**						
Smoking extent	0.087 [−0.125 to 0.300]	0.823	0.414	−0.196 [−0.414 to 0.023]	−1.791	0.079
EPIF	0.415 [0.199 to 0.630]	3.848	< 0.001	0.010 [−0.213 to 0.232]	0.086	0.932
CPZ EKVI	−0.269 [−0.462 to −0.076]	−2.796	0.007	−0.099 [−0.298 to 0.099]	−1.003	0.320

EPIF, estimated premorbid intellectual functioning. CPZ EKVI, antipsychotic medication dosage in chlorpromazine equivalents. All predictors were entered into the model as z-scores. ^a^Model 1: *F*(1, 59) = 1.662, *p* = 0.202, *R*^2^ = 0.027; Model 2: *F*(3, 57) = 9.320, *p* < 0.001, *R*^2^ = 0.329; Δ*R*^2^ = 0.302, *p* < 0.001. ^b^Model 1: *F*(1, 59) = 2.730, *p* = 0.104, *R*^2^ = 0.044; Model 2: *F*(3, 57) = 1.246, *p* = 0.302, *R*^2^ = 0.062; Δ*R*^2^ = 0.017, *p* = 0.594.

#### Impulsivity

Impulsivity component scores tended to decrease as cigarette smoking extent increased, but the relationship was not significant (Figure 2B). Covariates did not improve the overall model fit. The effects of them and the number of cigarettes smoked per day were non-significant (Table 4).

## Discussion

The cognitive self-medication hypothesis seeks to explain the high prevalence of smoking in schizophrenia. It argues that people with schizophrenia smoke to compensate for the cognitive and attentional symptoms of the disorder (Winterer, 2010). The hypothesis has attained widespread popularity, but the empirical evidence for it has been indirect or inconsistent (Ziedonis et al., 2008; Manzella et al., 2015). This study aimed to test predictions derived from the hypothesis, while also addressing methodological gaps that may have contributed to the heterogeneity of previous findings. We selected one of the most commonly used tests of smoking-related attentional changes, the CPT, and opted for its X-type variant (Conners, 2000). Following a principal component analysis of six CPT-X scores, we compared the performance of 61 smokers and 61 non-smokers with recent-onset SSDs, and additionally analyzed the smokers’ performance in relation to the extent of their habit (i.e., the number of cigarettes smoked per day). We also considered the impact of extraneous variables, including antipsychotic medication dosage and estimated premorbid intellectual functioning. The key finding from this study is that smokers with SSDs can demonstrate an attentional benefit as long as these covariates are controlled. However, the benefit is partial, modest, and apparently independent of the extent of the smoking behavior.

One of the previously proposed reasons for the inconsistency of findings on CPT performance in smokers with SSDs concerns the methods of CPT score analysis (Egeland and Kovalik-Gran, 2010a; Hahn et al., 2012). Fortunately, the sample size in this study was sufficient for a principal components analysis of the collected scores. Our analysis corroborated that no single construct underlined CPT performance (Conners, 2000; Egeland and Kovalik-Gran, 2010a; Conners, 2014). Instead, two components were extracted. One, referred to as impulsivity, received its highest loadings from errors of commission, detectability, and fast RTs. The other, labeled as inattention, received loadings from errors of omission, variability, and perseverations. This structure largely matched the one previously observed by Egeland and Kovalik-Gran (2010a) in a mixed sample of healthy controls and psychiatric patients. Like the authors of that study, we observed ambiguity in the CPT’s internal structure. In particular, perseverations contributed the strongest to the inattention component, despite being considered an indicator of impulsivity in normative data (Conners, 2000). Additionally, while fast RTs were strong indicators of impulsivity, slow RTs tended toward the inattention component in the unrotated solution. These findings suggest that the CPT’s internal structure may be diagnosis-specific. Indeed, previous factor analyses yielded different solutions in ADHD compared to other groups (Egeland and Kovalik-Gran, 2010b). The structural ambiguity and potential diagnosis-dependence of the CPT-X underscore the usefulness of deriving sample-specific component scores.

Impulsivity component scores appeared unaffected by smoking. The non-significant tendency was for smokers to perform worse than non-smokers. Similarly, a mildly negative association could be observed between cigarette smoking extent and impulsivity, but this tendency also failed to reach significance. All the outcomes have been previously reported for impulsivity-related scores, including them being better, same, or worse in smokers compared to non-smokers with SSDs (Zabala et al., 2009; Segarra et al., 2011; Wing et al., 2011; Hahn et al., 2012; Roth et al., 2013). The results of the current study favor the idea that individuals with SSDs show no smoking-related changes in impulsivity. The fact that this study used the CPT-X strengthens that conclusion. Due to its low signal-to-noise ratio, the CPT-X is considered a more sensitive measure of impulsivity than other variants such as the CPT-IP (Riccio et al., 2002; Roth et al., 2013), which other studies have utilized comparatively more frequently.

The inattention component showed evidence of a modest, smoking-related benefit. It has been suggested that the pertinent scores of variability (Wing et al., 2011) and omission rate (Zabala et al., 2009; Segarra et al., 2011) improved in smokers with SSDs. One study contested that: compared to non-smokers, smokers with SSDs recorded an elevated rate of errors of omission on the CPT-IP and a comparable rate on the CPT-X (Roth et al., 2013). While smokers did show signs of improved inattention in our study, the significance of this relationship emerged only upon the adjustment for estimated premorbid intellectual functioning and antipsychotic medication dosage. This suggests that insufficient control over intervening variables may have contributed to the previously reported inconsistencies. Indeed, smoking status alone only explained 0.1% of the variance observed in the inattention component score. The addition of covariates resulted in a model that explained an additional 25.4% of this variance. Both covariates contributed approximately equally to inattention in both groups. Higher estimates of premorbid intellectual functioning related to higher component scores, while higher antipsychotic medication dosage related to poorer scores. Although inverse relationships between antipsychotic medication dosage and cognitive performance have been documented previously, it is important to note that these are not necessarily causal, since they may be confounded by other factors such as overall illness severity or adverse metabolic effects (Hori et al., 2006; MacKenzie et al., 2018).

Interestingly, subsequent within-group analyses indicated that cigarette smoking extent and inattention were not related. The number of cigarettes smoked per day remained a non-significant predictor of inattention even upon including the previously favorable covariates, which did maintain statistical significance themselves. This suggested that the aforementioned smoking-related benefit was independent of the actual amount of nicotine consumed. To our knowledge, a similar difference between the effects of smoking status and extent has not yet been demonstrated. However, existing data reveal some support for it. For example, *ad-libitum* smoking appears to be associated with greater attentional benefits in participants with SSDs compared to controls (Wing et al., 2011), yet the groups respond comparably to the acute effects of nicotine (Barr et al., 2008). Also, overnight nicotine abstinence does not foster significant attentional impairments in SSDs, even though it does in controls (Beck et al., 2015). Finally, former smokers with SSDs outperform never-smokers with the disorders (Wing et al., 2011). Like the dose-independence we observed, these findings raise potential validity questions, i.e., they indicate that in SSDs, smoking status may be measuring something more than its name suggests.

We recommend that future studies explore additional variables that have so far been neglected, and that could more fully explain the smoking-related attentional benefits in SSDs. Novel explanations of the elevated prevalence of smoking in SSDs may also be needed. Present alternatives include other variants of the self-medication hypotheses, such as the idea that individuals with schizophrenia smoke to alleviate non-cognitive symptoms or medication side effects (Winterer, 2010; Manzella et al., 2015). However, these alternatives have also been disputed (Winterer, 2010; Manzella et al., 2015), and the present study has not gathered much support for them either. Smoking-related variables were independent of most of the clinical characteristics of our sample, including: diagnosis; duration of untreated psychosis; positive, negative, and general symptom severity; overall illness severity; and general functioning. The dosage of antipsychotic medication was higher in smokers compared to non-smokers. Smoking might counteract the effects of some types of antipsychotic medication, which could result in a need for increased medication dosage to achieve the targeted effect (Winterer, 2010). Nonetheless, given that the participants were recruited into this study after their first episode of psychosis, it remains unlikely that antipsychotic medication and its side effects were the cause of their smoking (see also Vaňurová, 2000).

A rebuttal of the cognitive self-medication hypothesis could have far-reaching clinical implications. The hypothesis is pervasive, and clinicians may hesitate to promote cigarette abstinence because of it (Ziedonis et al., 2008; Manzella et al., 2015). In a report for the National Institute of Mental Health, United States, Ziedonis et al. (2008: 1691) cautioned against using concepts of self-medication” to rationalize allowing ongoing tobacco use and limited smoking cessation efforts in many mental health treatment settings.” As the authors further illustrated, smoking in psychiatric facilities may be not only passively accepted but also implicitly encouraged: the habit provides opportunities for social contact among inpatients without the clinicians intruding on them. While the authors concur that motivation to quit smoking is generally lower among SSD patients, they also highlight that smoking cessation programs or nicotine replacement therapy can be effective in this population if available. However, in psychiatric settings, such programs continue to be rare (Kagabo et al., 2020).

It is of note that patients with schizophrenia often cite improved concentration among their reasons for smoking in self-report studies (Manzella et al., 2015). Even if this turned out to be unsubstantiated by objective measures, it could reduce a patient’s motivation to partake in smoking cessation programs (Ziedonis et al., 2008). Participation could be encouraged *via* adjunct therapies that help improve or maintain the client’s cognitive functioning levels. Psychosocial treatments could be among these. Psychosocial treatments support continued education, employment, everyday life functioning, social ability, or leisure activity. These and other factors, including premorbid intellectual functioning, can form the so-called cognitive reserve. The concept of the reserve was initially developed to explain the difference between pathology on the neural level and the clinical manifestation of a disorder (Stern, 2002). In SSDs, higher cognitive reserve relates to better functioning in most cognitive domains, including attention (Herrero et al., 2020; Rodriguez et al., 2022). Accordingly, estimated premorbid intellectual functioning was significantly associated with inattention in this study as well. The score was also significantly reduced in smokers compared to non-smokers. This was expected based on previous research (Ziedonis et al., 2008; Wing et al., 2011; Hidese et al., 2019). In fact, it has been suggested that lower premorbid intellect might contribute to the increased prevalence of smoking in SSDs by increasing the vulnerable individuals’ distress, which they then attempt to ameliorate by tobacco use (Hidese et al., 2019). The lower estimate of premorbid intellectual functioning in smokers with SSDs also highlights the need for rehabilitation. Rehabilitations that enhance cognitive reserve components could represent a healthier and more effective means of improving cognitive or general functioning in people with SSDs (Nuechterlein et al., 2008; Mueser et al., 2013). Cognitive remediation therapy could also be offered, as it can lead to robust improvements in several cognitive domains and overall psychosocial functioning (McGurk et al., 2007; Wykes et al., 2011).

### Limitations and future directions

The findings of this study should be interpreted in light of several limitations, including the narrow account of participant smoking history. Variables such as the nicotine content of a preferred tobacco product, time since last tobacco use, or lifetime duration of tobacco use were not measured in this study. Smoking history was also self-reported and not objectively affirmed. Since this was a cross-sectional study, we were unable to draw causal inferences from our results. Psychiatrically healthy controls were not included in this study, meaning that we could not address the comparative effects of smoking in participants with and without SSDs. Finally, smokers and non-smokers were unmatched with respect to their gender, with men being smokers more frequently than not. Fortunately, this limitation was unlikely to affect the results of this study, which derived from age- and sex-adjusted *T*-scores.

We believe that future research should focus on further testing of the cognitive self-medication hypothesis. Foremost, we suggest the replication and expansion of the present findings. Future studies may wish to collect a wider range of CPT scores related also to sustained attention and vigilance, or to investigate the applicability of the present results to a newer version of the CPT-X (Conners, 2014). Recruitment of a larger sample would also be beneficial, as it could increase statistical power, improve sampling adequacy, or even allow for a confirmatory factor analysis approach. It would also be beneficial to estimate premorbid functioning using more conventional or all-encompassing measures, such as the National Adult Reading Test (Nelson and Willison, 1991), or the Cognitive Reserve Assessment Scale in Health questionnaire (Amoretti et al., 2019), respectively. Additional intervening or mediating variables could also be explored. For example, it has been reported that smoking can decrease visual sensitivity (Fernandes et al., 2018). This could conceivably mask smoking-related benefits in visual attention tasks, such as the CPT. It may also be interesting to consider participants’ use of other substances that could affect visual attention, such as cannabis (Hájková et al., 2021; Setién-Suero et al., 2022).

Finally, we recommend investigating the temporal progression of smoking-related changes in attention. A prospective study of the high-risk population could be especially beneficial in this respect, as it would allow researchers to trace the tentative relationship between change in smoking behaviors and change in attentional performance. This has previously been done for non-cognitive symptoms and quality of life (Vermeulen et al., 2019), as well as for social cognition (Dekker et al., 2021). In both studies, smoking cessation did not affect the dependent variables, but whether this would also be true for cognitive and attentional performance remains to be seen.

## Conclusion

This study demonstrated that smokers with recent-onset SSDs can outperform non-smokers with the disorders on some attentional measures. Smokers showed better inattention scores insofar as the effects of antipsychotic medication dosage and estimated premorbid functioning were held constant. However, smoking explained a relatively small proportion of variance in inattention. Additionally, the extent of the participant’s smoking was unrelated to the inattention scores. No significant relationships were observed between smoking and impulsivity scores. In conclusion, although smoking status was a statistically significant predictor of some aspects of attentional performance in SSDs, its practical utility remained questionable. Future research should focus on further testing of predictions derived from the cognitive self-medication hypothesis. In the meantime, we recommend continued efforts on smoking cessation programs for clients with SSDs.

## Data availability statement

The raw data supporting the conclusions of this article will be made available by the authors, without undue reservation.

## Ethics statement

The studies involving human participants were reviewed and approved by the Research Ethics Board of the National Institute of Mental Health (Etická komise Národního ústavu duševního zdraví). The patients/participants provided their written informed consent to participate in this study.

## Author contributions

BK analyzed the data and wrote the manuscript. MR was involved in the conceptualization of the study and the acquisition of its funding, and also supervised the article’s writing. BK, KK, PŠ, and JJ collected the data. MR, KK, AS, AH, and MV reviewed and commented on the manuscript. All authors contributed to the article and approved the submitted version.

## Funding

This study is a result of research funded by the Czech Science Foundation as the GA ČR project no. 21-03615S “The Association between Cognition and Cognitive and Brain Reserve in First-Episode Schizophrenia Spectrum Disorders: A Prospective Study” and the GA ČR project no. 18-03125S “Prevalence of High-Risk Symptoms for Psychosis and their Relationship to Neurocognitive Performance and Daily Functioning in Population of Adolescents.” This work was also supported by the Czech Operational Programme “Research, Development and Education” (grant name: PharmaBrain; grant number: CZ.02.1.01/0.0/0.0/16_025/0007444).

## Conflict of interest

The authors declare that the research was conducted in the absence of any commercial or financial relationships that could be construed as a potential conflict of interest.

## Publisher’s note

All claims expressed in this article are solely those of the authors and do not necessarily represent those of their affiliated organizations, or those of the publisher, the editors and the reviewers. Any product that may be evaluated in this article, or claim that may be made by its manufacturer, is not guaranteed or endorsed by the publisher.
